# Realization of Joints of Aluminosilicate Glass and 6061 Aluminum Alloy via Picosecond Laser Welding without Optical Contact

**DOI:** 10.3390/ma17174299

**Published:** 2024-08-30

**Authors:** Caiwang Tan, Xing Lu, Fuyun Liu, Wei Song, Guanghui Guo, Qige Li, Yuhang Liu, Jianhui Su, Xiaoguo Song

**Affiliations:** 1 State Key Laboratory of Precision Welding & Joining of Materials and Structures, Harbin Institute of Technology, Harbin 150001, China; tancaiwang@hitwh.edu.cn (C.T.); 18487656415@139.com (X.L.); 18903298086@163.com (Q.L.); 18748783099@163.com (Y.L.); sujianhui0617@163.com (J.S.); songxg@hitwh.edu.cn (X.S.); 2 School of Materials Engineering, Shandong Institute of Shipbuilding Technology, Weihai 264209, China; 3 School of Mechanical & Electrical Engineering, Xuzhou University of Technology, Xuzhou 221018, China; swingways@hotmail.com; 4 CRRC Academy (Qingdao), Qingdao 266109, China; 15806599786@163.com

**Keywords:** aluminosilicate glass, 6061 aluminum alloy, high-power picosecond pulsed laser, non-optical contact, mechanical property

## Abstract

To achieve laser direct welding of glass and metal without optical contact is hard, owing to the large difference in thermal expansion and thermal conductivity between glass and metal and an insignificant melting area. In this study, the high-power picosecond pulsed laser was selected to successfully weld the aluminosilicate glass/6061 aluminum alloy with a gap of 35 ± 5 μm between glass and metal. The results show that the molten glass and metal diffuse and mix at the interface. No defects such as microcracks or holes are observed in the diffusion mixing zone. Due to the relatively large gap, the glass collapsed after melting and caulking, resulting in an approximately arc-shaped microcrack between modified glass and unmodified glass or weakly modified glass. The shape of the glass modification zone and thermal accumulation are influenced by the single-pulse energy and linear energy density of the picosecond laser during welding, resulting in variations in the number and size of defects and the shape of the glass modification zone. By reasonably tuning the two factors, the shear strength of the joint reaches 15.98 MPa. The diffusion and mixing at the interface and the mechanical interlocking effect of the glass modification zone are the main reasons for achieving a high shear strength of the joint. This study will provide reference and new ideas for the laser transmission welding of glass and metal in the non-optical contact conditions.

## 1. Introduction

Glass/metal connection has been widely applied in fields such as aerospace, optical sensing, and micro-electro-mechanical systems [1]. However, the large differences in physical and chemical properties between glass and metal make bonding difficult. With traditional connection methods such as cementation [2,3,4], mechanical connection [5], anodic bonding [6,7], diffusion bonding [8], and brazing [9,10] it is difficult to achieve efficient and reliable connection of the two materials. There are shortcomings such as aging, degassing, low bonding efficiency, and poor precision which restrict the further application of the connection of glass and metal.

In recent years, pulsed lasers have been rapidly developed and applied. Especially, picosecond (ps) and femtosecond (fs) ultrafast lasers provide a new powerful tool for transparent brittle materials. Based on the nonlinear absorption effect [11], a precise and firm connection between transparent brittle materials such as glass could be achieved via ps and fs ultrafast lasers. In addition, ps and fs ultrafast laser welding, considered a cold working process, results in a small heat-affected zone and high processing accuracy [12]. Based on this cold processing, a direct connection of glass and metal, which have significantly different physical properties like thermal expansion and thermal conductivity, can be achieved with ps and fs ultrafast lasers [13,14].

In current research on ps and fs ultrafast laser welding of glass and metal, optical contact is considered necessary [15]. Under optical contact conditions, the plasma produced via laser irradiation is limited in the interaction zone. Thus, most laser energy is absorbed by the interaction zone, resulting in a higher shear strength of the joint [16]. Zhan et al. [16] utilized an fs laser to weld fused silica and aluminum under optical contact conditions. The shear strength of the joint reached 25.75 MPa. Robert et al. [1] obtained the silica/copper, zerodur/invar, sapphire/titanium, and silica/aluminum joints with an fs laser under similar optical contact conditions. Among them, the joint shear strength of zerodur/invar and sapphire/titanium reached about 24 MPa. The aforementioned studies suggest that under the optical contact conditions, the glass and metal can be joined with high strength via ps and fs ultrafast laser welding. However, achieving optical contact conditions between glass and metal is extremely difficult. Generally, in order to obtain optical contact between glass and metal, the surfaces of materials usually need to be finely ground and polished, and the gap between glass and metal is controlled to less than λ/4 (λ is the wavelength of the laser) under a high external pressure [17,18]. Therefore, some studies on ps and fs ultrafast laser welding of glass and metal under non-optical contact conditions have been conducted.

The gaps in the non-optical contact conditions exist within a large range from a few micrometers to tens of micrometers. Some are small gaps of about 3 μm [19,20,21] and some are large gaps of tens of micrometers [15,22,23]. For small gap conditions, the surface treatment requirements for materials are less stringent than those under optical contact conditions. However, relatively simplified treatment procedures, including grinding and polishing material surfaces, are required to achieve the desired roughness and smoothness of the material surface. Therefore, it is not suitable for the connection of larger or longer pieces. Carter et al. [21] utilized a ps laser to weld square SiO_2_ and BK7 with a side length of 10 mm to aluminum alloy when the metal surface was still optically rough, and the joint strength of BK7 and aluminum alloy reached 13 MPa.

For the large gap conditions, there are no strict surface treatment requirements for glass and metal, making it suitable for specimens of different sizes. However, in current research, the mechanical properties of the joint are lower under large gap conditions. Ji et al. [15] realized fs laser welding of soda lime glass and Kovar alloy in large gap conditions, and the shear strength of the joint was only 2 MPa. Zhang et al. [23] realized fs laser welding of aluminosilicate glass and metal (aluminum, copper, and steel) without auxiliary pressure to control the gap between glass and metal. The shear strength of the aluminosilicate glass/copper joint was only 2.34 MPa. Therefore, exploring ways to enhance the mechanical properties of joints in glass and metal during ps and fs ultrafast laser welding under large gap conditions, and delving deeply into the connection mechanism, is necessary.

To melt more parent material to fill the large gap between glass and metal, a laser with sufficient power is required. However, because femtosecond pulses are extremely short, even with pulse energy measured in microjoules, the peak power of the pulse can reach the tens of megawatts (MW). This leads to significant nonlinear effects during transmission, which can either damage optical components or affect the characteristics of the pulse itself. Therefore, it is more challenging for femtosecond lasers to achieve high average power and high pulse energy [24,25]. Consequently, picosecond lasers are more suitable for welding glass and metal under conditions of large gaps. Additionally, for femtosecond pulses, mode-locking is almost the only method of implementation, but for picosecond pulses, especially those around 10 picoseconds, they can be directly obtained from semiconductor lasers using gain-switching technology. Thus, picosecond lasers are relatively more cost-effective [24,25].

In this study, the aluminosilicate glass and 6061 aluminum alloy (6061 Al) were welded directly with a high-power ps laser without any surface treatment. The effects of ps laser single-pulse energy and linear energy density on the macroscopic morphology and mechanical properties of the aluminosilicate glass/6061 Al joint were investigated. The connection mechanism of the aluminosilicate glass/6061 Al joint was deeply explored. The proposed method of aluminosilicate glass/6061 Al ps laser welding under large gap conditions could provide a cost-effective strategy for direct connection of transparent hard materials and metals.

## 2. Experimental

**Figure 1:**
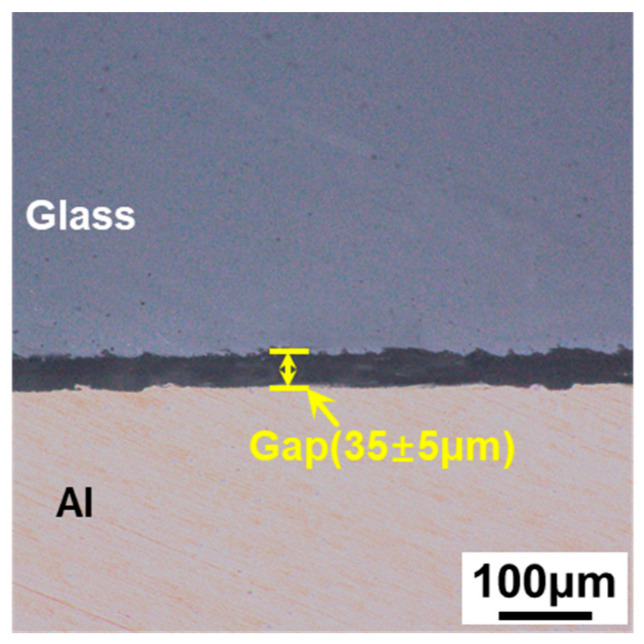
The size of the gap between glass and metal.

The commercial Corning Gorilla 5 aluminosilicate glass and 6061 aluminum alloy were used in this study with dimensions of 50 mm × 30 mm × 1 mm. The chemical compositions of the two materials are listed in Table 1 and Table 2 [26,27], respectively. Some physical properties of the two materials are listed in Table 3. Before welding, the glass and metal surfaces were treated using the ultrasonic cleaner to simply clean dust and oil. Figure 1 shows the gap between the glass and metal. In order to obtain the size of the gap, the gap between the glass and the metal was measured at intervals of 50 μm, and it was found that the size was 35 ± 5 μm.

**Figure 2:**
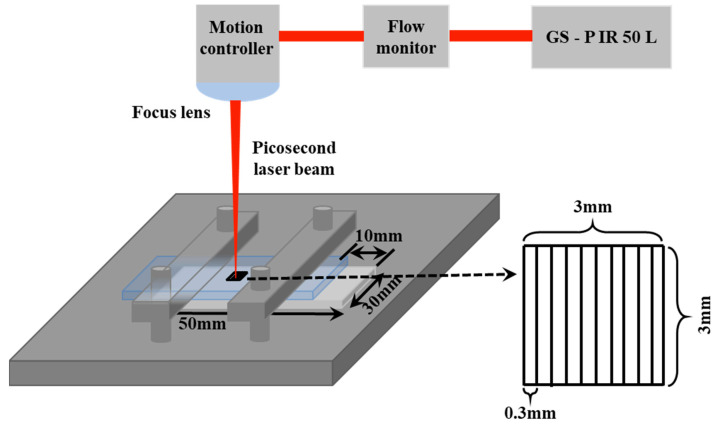
Schematic diagram of ps laser welding of aluminosilicate glass/6061 Al and the laser scanning paths.

Figure 2 shows the schematic diagram of ps laser welding of aluminosilicate glass/6061 Al and the laser scanning paths. The size of the welding area is 3 mm × 3 mm, and the row spacing is 0.3 mm. The aluminosilicate glass was assembled on the top of the 6061 Al. A high-power pulsed fiber ps laser (GS–PIR50L, Shenzhen United Winners Laser Co., Ltd., Shenzhen, China) with a waveform of 1064 nm, a maximum average output power of 50 W, and a repetition rate of 500~2000 kHz was selected for the heating source in this study. During the ps laser welding, the ps laser beam passes through the upper aluminosilicate glass and irradiates on the contact interface of the aluminosilicate glass and 6061 Al. The focusing method used in the ps laser devices is lens focusing, so the focal plane is fixed. In order to achieve focusing, the height of the carrier needs to be adjusted so that the glass–metal interface is in the focal plane.

The ps laser frequency and scanning speed correspond to the single-pulse energy and laser energy input, respectively. The laser output power corresponds to both of these factors. The single-pulse energy can be calculated via Equation (1).

(1) E=P/F

Here *E* is the single-pulse energy, *P* is the laser output power, and *F* is the frequency. The laser energy input corresponding to different scanning speeds and laser output power in this study is standardized according to the linear energy density, which is given by Equation (2).

(2) $$E_{a}=N\times P/V$$

Here *E_a_* is the linear energy density, *N* is the number of laser scans, *V* is the laser scanning speed, and *P* is the laser output power.

Table 4 lists the laser welding parameters used in this study and their corresponding linear energy density and single-pulse energy.

After welding, the macroscopic morphology and the cross-sectional morphology of the joint was analyzed with an optical digital microscope (OM, OLYMPUS DSX510, OLYMPUS, Shinjuku City, Japan). The microstructure and phase components of the ps laser welded joint were analyzed using a field emission scanning electron microscope (SEM, Quanta 200FEG, ZEISS-MERLIN Compact, Oberkochen, Germany) equipped with energy dispersion spectroscopy (EDS).

**Figure 3:**
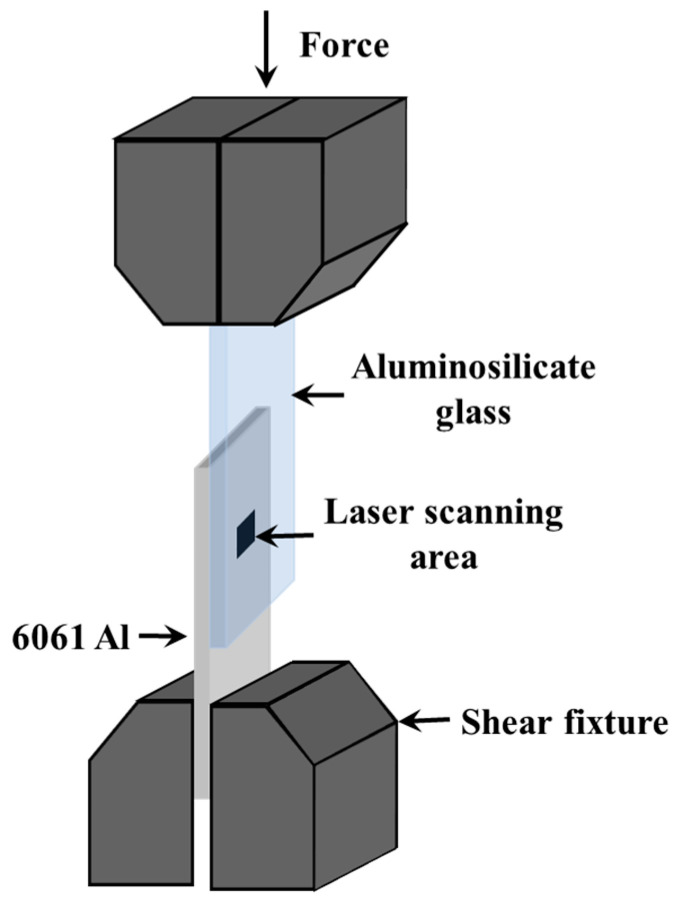
Schematic diagram of the fixture for shear strength tests.

The shear strength of the welded joint was tested using a universal tester (UTM, Instron 5967, Instron, Norwood, MA, USA) with a 0.2 mm/min compression speed. Figure 3 displays the schematic diagram of the fixture for shear strength testing. The specimen adopted the form of a lap joint. During the shear test, one chuck of the tensioner held one end of the specimen, and the other chuck was closed to slowly press down on the other end of the specimen. The joint shear strength is calculated with Equation (3).

(3) τ=F/S

where *τ* is the shear strength (MPa) of the joint, *F* is the fracture load (N) measured in the shear test, and *S* is the preset laser scanning area (mm^2^).

Three samples were tested for each set of parameters in the shear strength test to determine the average strength value. After the shear strength test, the fracture surface morphologies of the aluminosilicate glass side and aluminum side were analyzed with the optical digital microscope and field emission scanning electron microscope.

## 3. Results and Discussion

### 3.1. Typical Macroscopic Morphology and Microstructure

**Figure 4:**
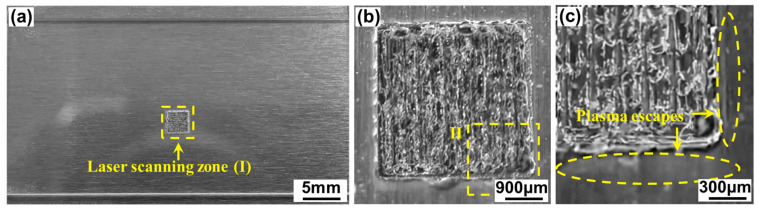
Typical macroscopic morphology of ps laser-welded aluminosilicate glass/6061 Al weld seams: (**b**) enlarged image of zone Ⅰ in (**a**); (**c**) enlarged image of zone Ⅱ in (**b**).

Figure 4 shows the typical macroscopic morphology of aluminosilicate glass/6061 Al weld seams via ps laser welding. The aluminosilicate glass/6061 Al is well joined and no obvious macrocracks or other macroscopic welding defects are observed, as presented in Figure 4a,b. However, at the laser scanning zone, the glass side is gray and white, and the track of the laser scan is not clear. This is because the glass side will collapse to fill the gap after melting due to the large gap between the aluminosilicate glass and 6061 Al. In addition, the thermal expansion and modification of aluminosilicate glass produce internal stress inside the glass, leading to the generation of microcracks in the glass. Moreover, some silvery gray dust is formed around the laser welding zone, as shown in Figure 4c. This is caused by part of the plasma produced via laser irradiation escaping from the interfacial gap. In laser welding, the plasma will be recombined and deposited back onto the surface of material under the action of gravity and atmospheric pressure [28].

**Figure 5:**
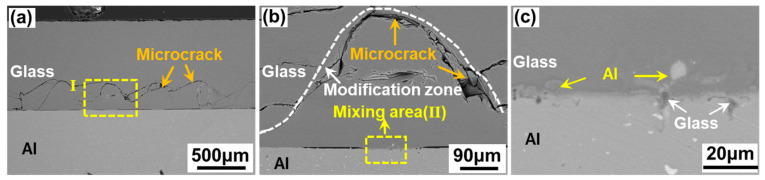
Typical cross-sectional morphology of aluminosilicate glass/6061 Al joints obtained via ps laser welding: (**b**) enlarged image of zone Ⅰ in (**a**); (**c**) enlarged image of zone Ⅱ in (**b**).

Figure 5 presents the cross-sectional morphology of the aluminosilicate glass/6061 Al ps laser-welded joints. The joint can be divided into four parts including the base metal, diffusion mixing zone, glass modification zone, and base glass, as shown in Figure 5a,b. The glass modification zone has the tendency to move away from the glass side. Therefore, an almost continuous, approximately arc-shaped microcrack formed between the glass modification zone and the glass base material. The generation of the microcracks can be attributed to two reasons. First, in the ps laser welding, the glass will be collapsed when the gap between glass and aluminum alloy is filled with the molten glass to form a connection. Thus, the approximately arc-shaped microcracks between the modified glass and the unmodified glass or the weakly modified glass will be generated. The glass modification zone, akin to being embedded in the surface on the glass side, offers a mechanical interlocking effect in shear tests. Second, in the ps laser welding, the internal stress inside the glass can be produced by the thermal expansion and modification of the glass. In this case, microcracks will also be generated on the glass side. The interface at the glass and aluminum is well joined via the ps laser welding without obvious pores and microcracks, as suggested in Figure 5c. There are some pits on the surface of the aluminum alloy and the pits are filled with glass. Part of the aluminum alloy moves into the glass, leading to the formation of larger particles. The glass and aluminum alloy at the interface diffuse and mix with each other, which can be caused by the recoil pressure produced by the thermolysis of the aluminum alloy under laser irradiation [29].

**Figure 6:**
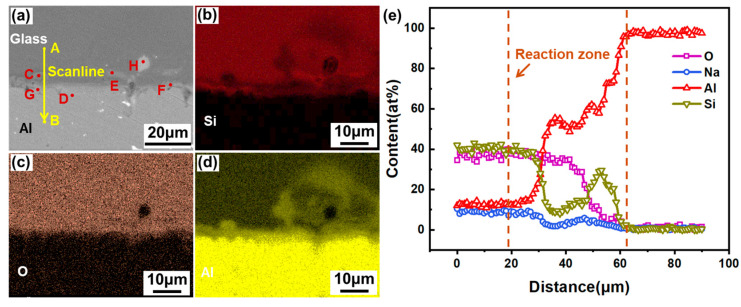
(**a**) Interface zone of aluminosilicate glass/6061 Al joint; (**b**–**d**) EDS surface scan of (**a**); (**e**) EDS line scan result across the interface along the yellow line marked in (**a**).

Figure 6 shows the EDS line scan and surface scan results of the interface zone in the aluminosilicate glass/6061 Al welded joint. Figure 6e is obtained via line scanning from point A to point B along the yellow arrow line in Figure 6a at a distance of 100 μm. Table 5 lists the EDS compositions of the microzones marked in the interfacial zone of the glass/Al welded joint. The Si, Al, and O diffuse with each other at the interface, as suggested in Figure 6b–d. Specially, the Al element has a longer diffusion distance, and part of the aluminum element moves to a deeper area of the glass side and is mixed with the glass. There is a reaction region of about 40 μm in the ps laser-welded joint interface of glass/Al, as seen in Figure 6e. In this region, from the glass side to the aluminum alloy side, the content of the Si element first decreases and then increases and then decreases again, while the content of the Al element has the opposite variation tendency. Therefore, there is mutual diffusion and mixing between both the glass and aluminum alloy at the interface. According to Table 5, the new phases will be produced at the interface, which are preliminarily identified as the Al–Si–O compound [30,31] and Al.

### 3.2. Effect of Single-Pulse Energy on Aluminosilicate Glass/6061 Al ps Laser Welding

#### 3.2.1. Effect of Single-Pulse Energy on Macroscopic and Cross-Sectional Morphology of Aluminosilicate Glass/6061 Al ps Laser Welding Joint

**Figure 7:**
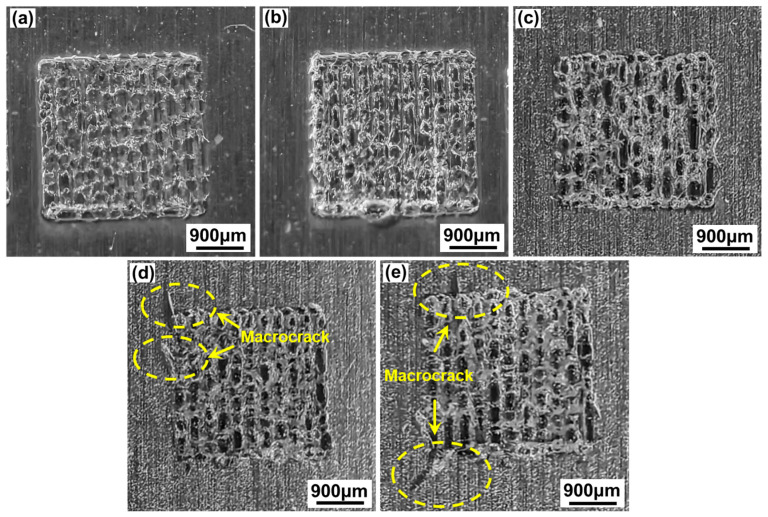
The effect of different levels of single-pulse energy with a linear energy density of 32 J/cm on the macroscopic morphologies of aluminosilicate glass/6061 Al joints: (**a**) 36.37 μJ; (**b**) 40 μJ; (**c**) 44.44 μJ; (**d**) 50 μJ; (**e**) 57.14 μJ.

Figure 7 shows the macroscopic morphologies of the aluminosilicate glass/6061 Al joint welded with a ps laser using different single-pulse energy levels ranging from 36.37 μJ to 57.14 μJ with a linear energy density of 32 J/cm. At the laser scanning zone, the glass side is gray and white, and the laser scanning track is blurry. With the increase in the single-pulse energy, the formation of the laser scanning zone gradually deteriorates. When the single-pulse energy is less than or equal to 40 μJ, the laser scanning zone is well formed, as shown in Figure 7a,b. However, when the single-pulse energy is greater than or equal to 50 μJ, the formation of the laser scanning zone is poor, and some macrocracks are formed in the corner of the laser scanning zone, as shown in Figure 7d,e. This is because many microcracks will be generated and extended due to large thermal stresses caused by the effect of the large single-pulse energy and strong heat accumulation. These microcracks coalesce to form macrocracks.

**Figure 8:**
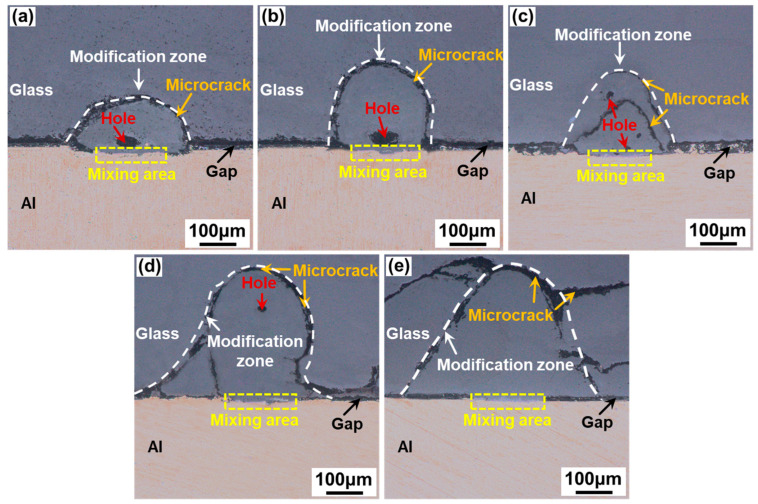
Cross-sectional morphology of aluminosilicate glass/6061 Al joints with different single-pulse energy levels with a linear energy density of 32 J/cm: (**a**) 36.37 μJ; (**b**) 40 μJ; (**c**) 44.44 μJ; (**d**) 50 μJ; (**e**) 57.14 μJ.

Figure 8 shows the cross-sectional morphology of aluminosilicate glass/6061 Al joints welded with a ps laser at different single-pulse energy levels. With the increase in the single-pulse energy, the area of the glass modification zone gradually increases, and the size and number of microcracks also increase. Moreover, when the single-pulse energy is lower than or equal to 50 μJ, the holes are formed in the glass modification zone. As shown in Figure 8a,b, almost no microcracks are produced, except for the approximately arc-shaped microcrack between the glass modification zone and the glass base material. In addition, the glass modification zone of Figure 8a is short and wide. When the single-pulse energy is between 44.44 μJ and 50 μJ, some microcracks are generated inside the glass modification zone, as presented in Figure 8c,d. Especially, in Figure 8c, another approximately arc-shaped microcrack is produced inside the glass modification zone. When the single-pulse energy is increased to 57.14 μJ, many microcracks inside the glass side are generated and extended due to the large thermal stresses caused by the large single-pulse energy and strong heat accumulation, as shown in Figure 8e.

**Figure 9:**
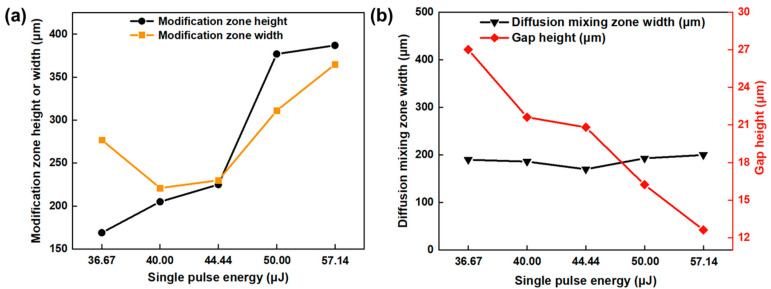
(**a**) Relationship between the modification zone height, modification zone width, and single-pulse energy; (**b**) relationship between the diffusion mixing zone width, gap height, and single-pulse energy.

Figure 9 presents the relationship between the glass modification zone height, glass modification zone width, diffusion mixing zone width, interface gap height, and the single-pulse energy. With the increase in the single-pulse energy, the modification zone height gradually increases, and the modification zone width first decreases and then increases, while the area of glass modification zone gradually increases. Because the generation of microcracks on the glass side, the transparency of the glass is reduced, which causes the laser input energy to accumulate in the glass side. In this case, with the increase in the single-pulse energy, the energy accumulation effect improves, leading to an increase in the area of the glass modification zone, as shown in Figure 9a. The diffusion mixing zone width is almost unchanged. This is because the laser energy input is constant due to the unvaried linear energy density. In addition, due to the solidification shrinkage effect of molten glass and metal, the more glass and metal melt, the smaller the gap that will be obtained. Thus, the interface gap gradually decreases when the single-pulse energy increases, as suggested in Figure 9b.

#### 3.2.2. Effect of Single-Pulse Energy on Mechanical Properties of Aluminosilicate Glass/6061 Al ps Laser Welding Joint

**Figure 10:**
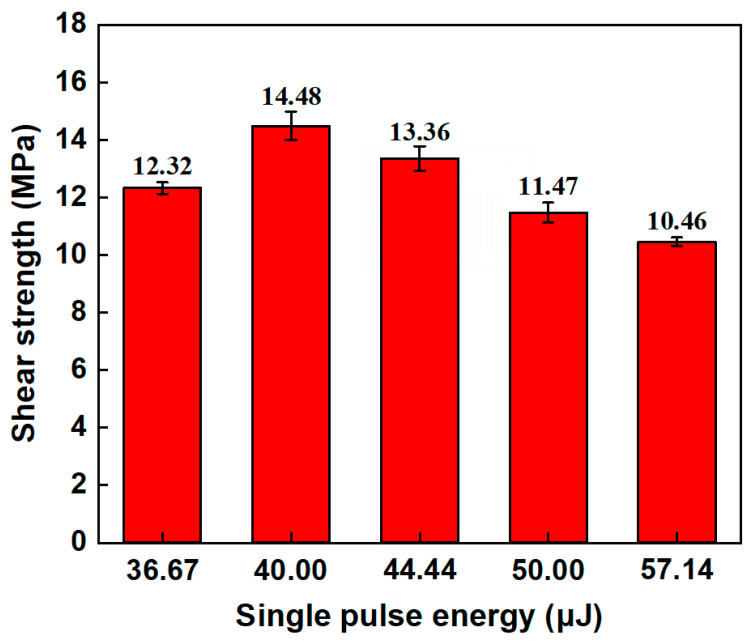
Shear strength of aluminosilicate glass/6061 Al joints with different single-pulse energy levels.

Figure 10 shows the shear strength of the aluminosilicate glass/6061 Al joints under different single-pulse energy levels. With the increase in the single-pulse energy, the shear strength of the joint is first increased and then decreased. The shear strength reaches a maximum value of 14.48 MPa when the single-pulse energy is 40 μJ. According to Figure 8, as the single-pulse energy decreases below 40 μJ, the area of the glass modification zone decreases, and the shape of the glass modification zone becomes shorter and wider, resulting in a reduction in its mechanical interlocking effect. In this case, the shear strength of the joint decreases. When the single-pulse energy exceeds 40 μJ, as the single-pulse energy increases, the area of the glass modification zone increases, enhancing the mechanical interlocking effect. However, the number and size of microcracks also increases, with some microcracks forming inside the glass modification zone, which can become a source of fracture, leading to a significant reduction in shear strength. Thus, the shear strength of the joint also decreases.

**Figure 11:**
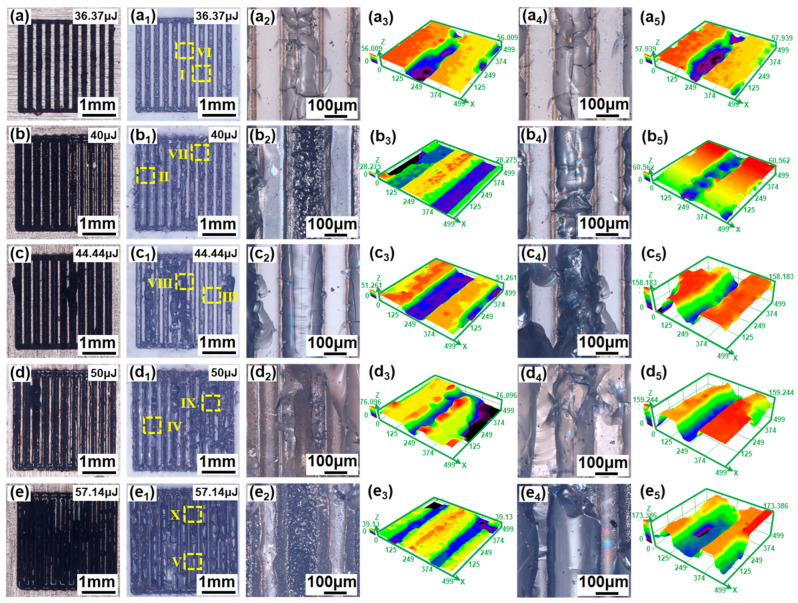
Optical digital microscope images of the fracture surface of aluminosilicate glass/6061 Al joints with a single-pulse energy between 36.37 μJ and 57.14 μJ: (**a**–**e**) The fracture surface morphology of the Al side; (**a_1_**–**e_1_**) the fracture surface morphology of the glass side; (**a_2_**–**e_2_**) enlarged image of zone Ⅰ~Ⅴ in (**a_1_**–**e_1_**); (**a_3_**–**e_3_**) the 3D morphology of (**a_2_**–**e_2_**); (**a_4_**–**e_4_**) enlarged image of zone Ⅵ~Ⅹ in (**a_1_**–**e_1_**); (**a_5_**–**e_5_**) the 3D morphology of (**a_4_**–**e_4_**).

Figure 11 shows the morphologies of fracture surfaces after shear strength tests of the aluminosilicate glass/6061 Al joints welded with the different single-pulse energy levels. When the single-pulse energy is 36.37 μJ, almost all weld seams are found to be fractured on the glass side, as presented in Figure 11(a–a_5_). This is attributed to a reduction in mechanical interlocking, which results from both the reduced area and the short and wide shape of the glass modification zone. When the single-pulser energy is increased to 40 μJ, some weld seams fracture on the glass side and some weld seams fracture at the interface, as shown in Figure 11(b–b_5_). This is because the mechanical interlocking effect is enhanced due to the appropriate size and shape of the glass modification zone. With the single-pulse energy continues to increase, almost all of the weld seams fracture again on the glass side, as suggested in Figure 11(c–c_5_). This is related to the microcracks generate within the glass modification zone and the not large area of the glass-modified zone. Moreover, when the single-pulse energy exceeds 20, some weld seams fracture on glass side and some weld seams fracture at the interface again, as shown in Figure 11(d–d_5_,e–e_5_). Although a larger area of the glass modification zone can provide a greater mechanical interlocking effect, the larger single-pulse energy and the greater thermal accumulation effect lead to the formation of more and larger microcracks. Therefore, when the single-pulse energy is high, not all weld seams fracture at the interface.

**Figure 12:**
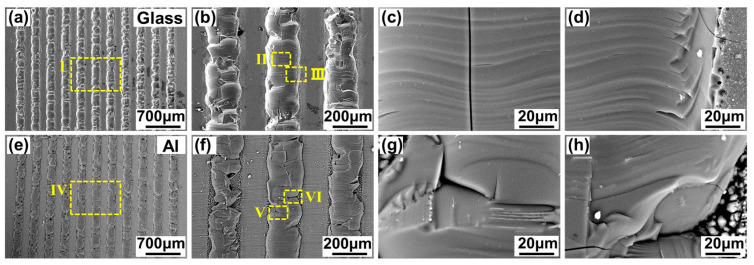
SEM image of the fracture morphology of aluminosilicate glass/6061 Al weld seams when the line energy density is 32 J/cm and the single-pulse energy is 36.37 μJ: (**a**) the fracture morphology on the glass side; (**b**) enlarged image of zone Ⅰ in (**a**); (**c**,**d**) enlarged image of zone Ⅱ~Ⅲ in (**b**); (**e**) the fracture morphology on the metal side; (**f**) enlarged image of zone Ⅳ in (**e**); (**g**,**h**) enlarged image of zone Ⅴ~Ⅵ in (**f**).

According to the above fracture surface analysis, two fracture patterns can be concluded. Figure 12 shows the SEM image of Figure 11(a,a_1_). This fracture pattern is named fracture pattern Ⅰ. This fracture pattern is of an obvious brittle fracture. All weld seams are fractured on the glass side. On the glass side, the glass of the weld seams is pulled out and adheres to the metal side. This is caused by the weaker mechanical interlocking effect of the glass modification zone.

**Figure 13:**
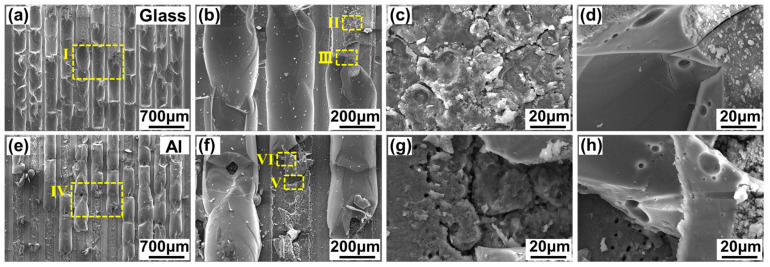
SEM image of the fracture morphology of aluminosilicate glass/6061 Al weld seams when the line energy density is 32 J/cm and the single-pulse energy is 57.14 μJ: (**a**) The fracture morphology on the glass side; (**b**) enlarged image of zone Ⅰ in (**a**); (**c**,**d**) enlarged image of zone Ⅱ~Ⅲ in (**b**); (**e**) the fracture morphology on the metal side; (**f**) enlarged image of zone Ⅳ in (**e**); (**g**,**h**) enlarged image of zone Ⅴ~Ⅵ in (**f**).

Figure 13 shows the SEM image of Figure 11(e,e_1_). This fracture pattern is named fracture pattern Ⅱ. Some weld seams fracture on the glass side and some weld seams fracture at the interface. The glass side is badly damaged. When the fracture occurs on the glass side, the fracture pattern is clearly of a brittle fracture, as shown in Figure 13d,h. When the fracture occurs at the interface, a number of dimples can be seen on the fracture surface, as shown in Figure 13c,g. Therefore, the fracture pattern is of a plastic fracture when fracture occurs at the interface. According to the fracture surface analysis, this fracture pattern is a mixture of brittle and plastic fractures.

### 3.3. Effect of Linear Energy Density on Aluminosilicate Glass/6061 Al ps Laser Welding

#### 3.3.1. Effect of Linear Energy Density on Macroscopic and Cross-Sectional Morphology of Aluminosilicate Glass/6061 Al ps Laser Welding Joint

**Figure 14:**
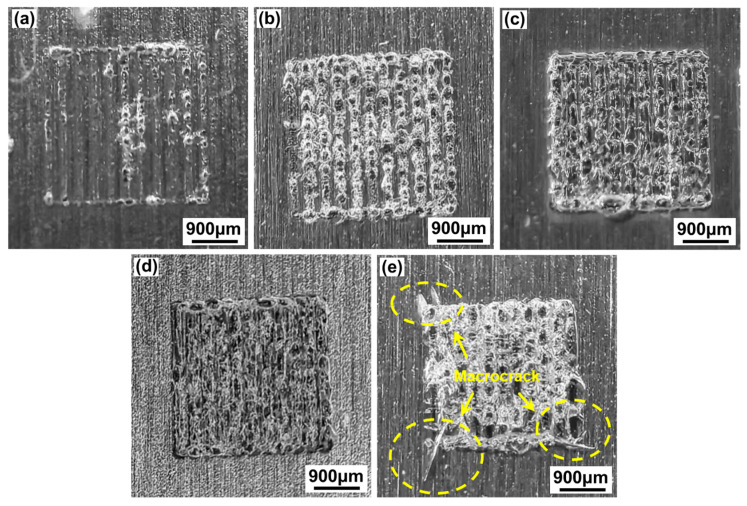
The effect of different linear energy densities with a single-pulse energy of 45 μJ on the macroscopic morphologies of aluminosilicate glass/6061 Al joints: (**a**) 22.86 J/cm; (**b**) 26.67 J/cm; (**c**) 32 J/cm; (**d**) 40 J/cm; (**e**) 53.33 J/cm.

Figure 14 shows the macroscopic morphologies of aluminosilicate glass/6061 Al joints welded with a ps laser using different linear energy densities ranging from 22.86 J/cm to 53.33 J/cm with a single-pulse energy of 40 μJ. When the linear energy density is less than or equal to 26.67 J/cm, the formation of the laser scanning zone gradually became better as the linear energy density decreases, as shown in Figure 14a,b. This is because when the line energy density is low, the metal and glass hardly melt due to the low laser energy input. When the linear energy density exceeds 26.67 J/cm, the formation of the laser scanning zone initially improves before deteriorating with further increases in linear energy density, as depicted in Figure 14b–e. This is attributed to the increase in the linear energy density, which leads to an increase in the amount of melted glass and metal, facilitating easier caulking with fewer defects. However, when the linear energy density is too large, the internal stresses inside the glass are high and more defects are produced due to the large laser energy input and the strong thermal accumulation effect.

**Figure 15:**
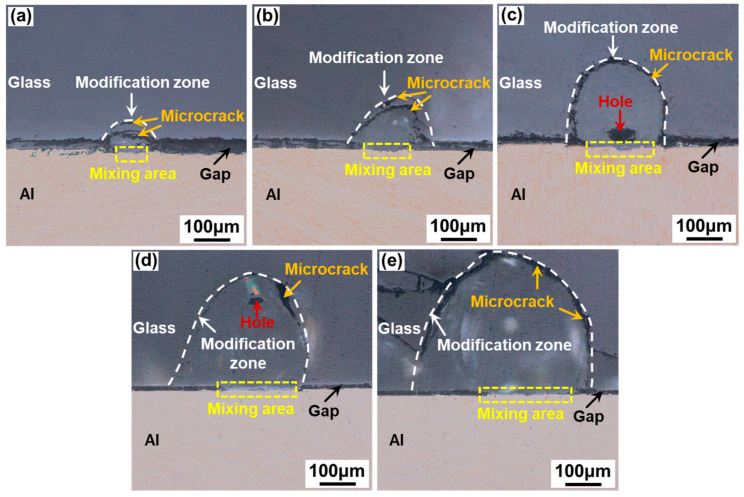
Cross-sectional morphology of aluminosilicate glass/6061 Al joints with different linear energy densities with a single-pulse energy of 40 μJ: (**a**) 22.86 J/cm; (**b**) 26.67 J/cm; (**c**) 32 J/cm; (**d**) 40 J/cm; (**e**) 53.33 J/cm.

Figure 15 shows the cross-sectional morphology of aluminosilicate glass/6061 Al joints welded with a ps laser at different linear energy densities. With the increase in the linear energy density, the area of the glass modification zone gradually increases. Moreover, when the linear energy density is between 32 J/cm and 40 J/cm, the holes are formed in the glass modification zone. As shown in Figure 15a,b, when the linear energy density is low, less glass and metal are melted, resulting in the creation of many microcrack defects after caulking, which are large in size relative to the glass modification zone. When the linear energy density exceeds 26.67 J/cm, the number and the relative size of microcracks gradually decrease due to the increase in molten glass, as indicated in Figure 15c,d. However, when the linear energy density is increased to 53.33 J/cm, many large microcracks are generated on the glass side due to the large internal stresses inside the glass caused by the large laser energy input and the large thermal accumulation effect, as suggested in Figure 15e.

**Figure 16:**
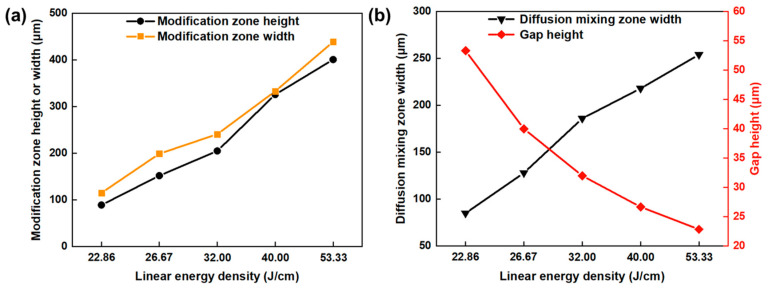
(**a**) Relationship between the modification zone height, modification zone width, and linear energy density; (**b**) relationship between the diffusion mixing zone width, gap height, and linear energy density.

Figure 16 presents the relationship between the glass modification zone height, glass modification zone width, diffusion mixing zone width, interface gap height, and the linear energy density. With the increase in the linear energy density, the modification zone height, modification zone width, and diffusion mixing zone width increase. These variations are attributed to the increase in the laser energy input, leading to the increase in molten glass and metal. Moreover, the interfacial gap has the opposite variation rule. This is because the solidification shrinkage effect of molten glass and metal. Therefore, the more the glass and metal melt, the smaller the gap will be.

#### 3.3.2. Effect of Linear Energy Density on Mechanical Properties Aluminosilicate Glass/6061 Al ps Laser Welding Joint

**Figure 17:**
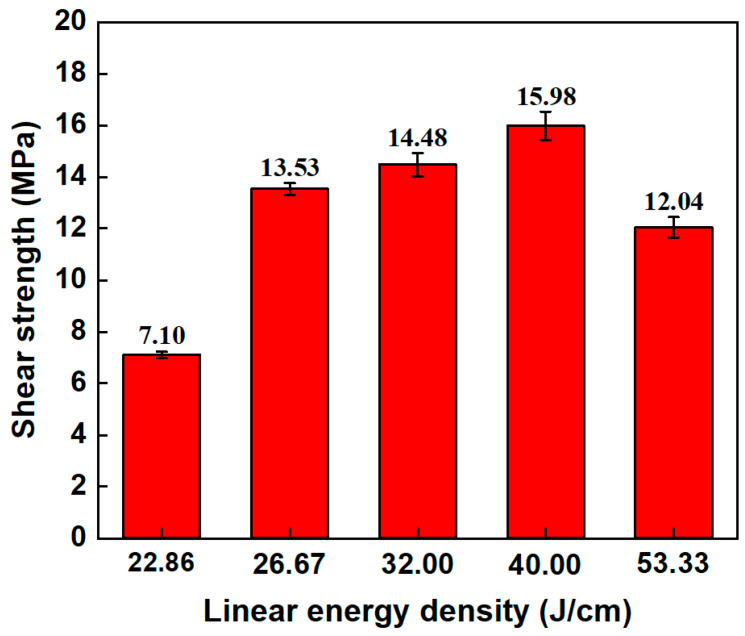
Shear strength of aluminosilicate glass/6061 Al joints with different linear energy densities.

Figure 17 shows the shear strength of the aluminosilicate glass/6061 Al joints under different linear energy densities. With the increase in linear energy density, the shear strength of the joint is increased first and then decreased. The shear strength reaches a maximum value of 15.98 MPa when the linear energy density is 40 J/cm. According to Figure 15, with the increase in the linear energy density, the molten glass increases, and the number of and relative size of microcracks decrease, resulting in improvement in the mechanical interlocking effect. Thus, the shear strength of the joint improves. However, when the linear energy is too large, the thermal stress will be higher. Therefore, a lot of large microcracks are generated within the glass side. Eventually, the shear strength of the joint declines.

**Figure 18:**
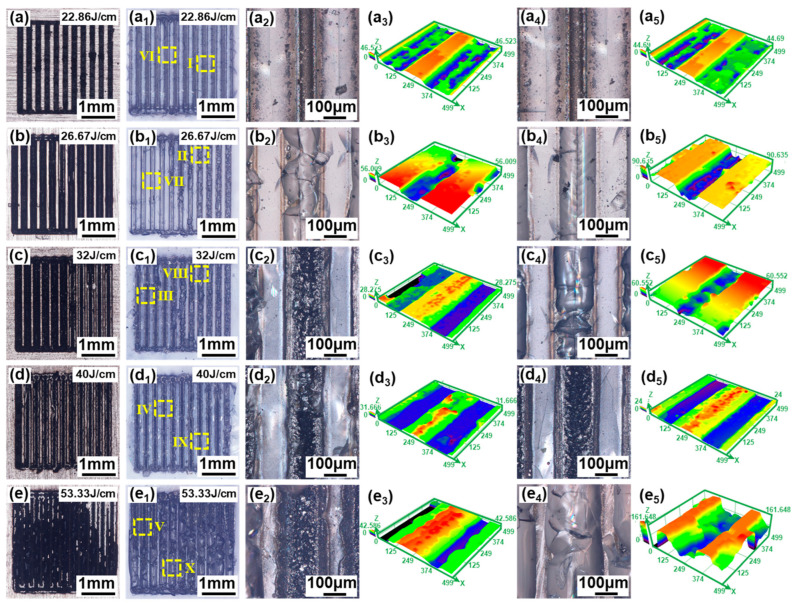
Optical digital microscope images of the fracture surface of aluminosilicate glass/6061 Al joints with a linear energy density between 22.86 J/cm and 53.33 J/cm: (**a**–**e**) the fracture surface morphology of the Al side; (**a_1_**–**e_1_**) the fracture surface morphology of the glass side; (**a_2_**–**e_2_**) enlarged image of zone Ⅰ~Ⅴ in (**a_1_**–**e_1_**); (**a_3_**–**e_3_**) the 3D morphology of (**a_2_**–**e_2_**); (**a_4_**–**e_4_**) enlarged image of zone Ⅵ~Ⅹ in (**a_1_**–**e_1_**); (**a_5_**–**e_5_**) the 3D morphology of (**a_4_**–**e_4_**).

Figure 18 shows the morphologies of fracture surfaces after shear strength tests of the aluminosilicate glass/6061 Al joints welded with different linear energy densities. When the linear energy density is less than or equal to 26.67 J/cm, the glass melting volume is smaller due to the low linear energy density, which leads to the generation of many relatively large microcracks and a decrease in the mechanical interlocking effect. Thus, all weld seams fracture on the glass side, as presented in Figure 18(a–a_5_,b–b_5_). With the increase in the linear energy density, the melting volume of glass increases, resulting in the generation of few and relatively small microcracks and a high mechanical interlocking effect. Therefore, some weld seams fracture on the glass side and some weld seams fracture at the interface, as shown in Figure 18(c–c_5_). When the linear energy density is increased to 40 J/cm, almost all weld seams fracture at the interface, as suggested in Figure 18(d–d_5_). This is attributed to the appropriate laser energy input resulting in more glass melting, which leads to the generation of fewer and relatively smaller microcracks and a higher mechanical interlocking effect. Therefore, almost all weld seams fracture at the interface. However, when the linear energy density increases further, the fracture surface morphology, with some weld seams fracturing on the glass side and others at the interface, appears again, as shown in Figure 18(e–e_5_). This is because the higher linear energy density leads to larger thermal stresses. Therefore, many large microcracks inside the glass are generated, which easily become fracture sources.

**Figure 19:**
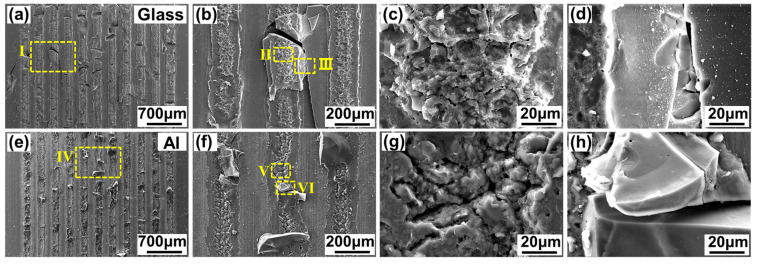
SEM image of the fracture morphology of aluminosilicate glass/6061 Al weld seams when the line energy density is 40 J/cm and the single-pulse energy is 40 μJ: (**a**) the fracture morphology on the glass side; (**b**) enlarged image of zone Ⅰ in (**a**); (**c**,**d**) enlarged image of zone Ⅱ~Ⅲ in (**b**); (**e**) the fracture morphology on the metal side; (**f**) enlarged image of zone Ⅳ in (**e**); (**g**,**h**) enlarged image of zone Ⅴ~Ⅵ in (**f**).

According to the fracture surface analysis, a new fracture pattern, in which all fractures occur at the interface, appears in addition to the two patterns discussed in Section 3.3.2. Figure 19 shows the SEM image of Figure 18(d,d_1_). This fracture pattern is named fracture pattern Ⅲ. Almost all weld seams fracture at the interface and few glass fragments adhere to the metal side. Both the glass side and the metal side exhibit a large number of dimples at the fracture surface. The fracture pattern is mainly plastic fracture with less brittle fracture, which is caused by the larger mechanical interlocking effect of the glass modification zone.

### 3.4. Student’s t Test and the Comparison between This Study and Previous Studies

#### 3.4.1. Student’s *t* Test

The Student’s *t*-test is a commonly used statistical test method to determine whether there is a significant difference between two sets of data, or whether the sample mean of a set of data is significantly different from the known population mean. The Student’s *t*-test is suitable for situations where the sample size is small (usually less than 30) and the data are normally distributed. Its calculation formula is as follows:

(4) $$t=\frac{\left|\bar{x_{1}}-\bar{x_{2}}\right|}{\sqrt{s_{1}^{2}/n_{1}+s_{2}^{2}/n_{2}}}$$

Here *t* is the result of Student’s *t*-test, $\bar{x_{1}}$ is the sample 1 mean, $\bar{x_{2}}$ is the sample 2 mean$,\;\;s_{1}^{2}$ is the sample 1 deviation, $n_{1}$ is the number of sample 1, $s_{2}^{2}$ is the sample 2 deviation, $n_{2}$ is the number of sample 2.

Student’s *t*-tests were performed on the shear strength of two groups of welded joints for single-pulse energy and line energy densities. The *t*-value of them is 0.022, which is less than the 0.05 threshold. Therefore, when these two laser parameters are adjusted, they lead to statistically significant differences in shear strengths.

#### 3.4.2. The Comparison between This Study and Previous Studies

**Figure 20:**
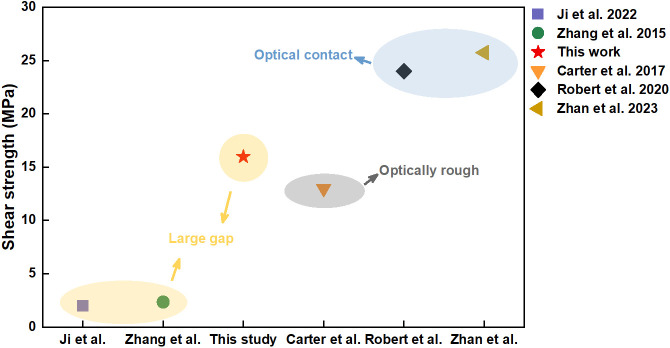
Comparison of mechanical properties between this study and previous studies: Ji et al. [15]; Zhang et al. [23]; Carter et al. [21]; Robert et al. [1]; Zhan et al. [16].

Figure 20 shows the comparation of mechanical properties between this study and previous studies. In previous studies, the shear strength of the ultrafast laser-welded joint of glass/metal under large gap conditions was about 2 MPa. When the material surfaces were optically rough, this strength increased to about 13 MPa. Moreover, under optical contact conditions, the shear strength of the ultrafast laser-welded joint of glass/metal reached about 25 MPa. In this study, the surfaces of materials were treated via a simple alcohol wash. A large gap existed between the glass and metal. In this condition, the glass and metal were welded with a ps laser, and the shear strength of the joint reached 15.98 MPa. This mechanical property significantly exceeds those reported for ultrafast laser-welded joints of glass and metal under large gap conditions in previous studies. It also exceeds the mechanical properties of ultrafast laser-welded joints of glass and metal when the material surfaces were optically rough. However, it is lower than the mechanical properties of ultrafast laser-welded joints of glass and metal under optical contact conditions. In conclusion, this study obtains a relatively excellent mechanical property under simple process treatment. It is an efficient and economical method of glass and metal ultrafast laser welding.

## 4. Conclusions

In this study, the joint connection mechanism of the ps laser-welded joint of aluminosilicate glass/6061 Al under non-optical conditions was explored. The effect of single-pulse energy and linear energy density on macroscopic morphology and mechanical properties was investigated. The conclusions are listed as follows.

(1) Aluminosilicate glass and 6061 Al were successfully welded with a ps laser at a gap of 30 ± 5 μm. The main reason for the formation of the connection is the diffusion mixing of molten glass and metal at the interface, consisting of the Al–Si–O compound and Al. The glass will collapse after melting and caulking, resulting in an approximately arc-shaped microcrack between the modified glass and the unmodified glass or the weakly modified glass due to the relatively large gap between glass and metal caused by the rough surface of material;
(2) Single-pulse energy and linear energy density affect the shape of the glass modification zone and thermal accumulation. The Student’s *t*-test, with a t-value of 0.022, indicates a statistically significant difference in shear strength when adjusting the two parameters. By properly adjusting these two parameters, the shear strength of the aluminosilicate glass/6061 Al joint reaches the maximum value of 15.98 MPa, which is excellent under the conditions of simple cleaning of the material surface only;
(3) By analyzing the morphologies of the fracture surfaces, three fracture patterns can be identified: fracture pattern I in which the fractures occur partly on the glass side and partly at the interface; fracture pattern II, where the fractures occur entirely on the glass side; and fracture pattern III, where the fractures occur entirely at the interface. Fracture pattern III exhibits the better joint macroscopic morphology and cross-sectional morphology, as well as the maximum joint shear strength.

## Figures and Tables

**Table 1:** Chemical composition list of aluminosilicate glass (wt. %).

Element	Si	Na	Ca	O	Al
Content	34.55–42.61	9.21	1.38	35.61–39.95	10.80–15.30

**Table 2:** Chemical composition list of 6061 Al (wt. %).

Element	Si	Fe	Cu	Mn	Mg	Cr	Zn	Ti	Al
Content	0.4–0.8	0.7	0.15–0.4	0.15	0.8–1.2	0.04–0.35	0.25	0.15	Bal

**Table 3:** Physical property parameters of experimental materials.

Material	Melting Point/°C	Density/g·cm^−3^	Thermal Conductivity/Wm^−1^k^−1^	Thermal Expansion Coefficient/10^−6^k^−1^
Aluminosilicate glass	1425	2.42	1.67	8.69
6061 Al	650	2.75	168	23.5

**Table 4:** The laser parameters selected in welding.

Number	Scanning Speed (mm/s)	Frequency (kHz)	Power (W)	Linear Energy Density (J/cm)	Single-Pulse Energy (μJ)
1	25	1100	40	32.00	36.37
2	25	1000	40	32.00	40.00
3	25	900	40	32.00	44.44
4	25	800	40	32.00	50.00
5	25	700	40	32.00	57.14
6	35	1000	40	22.86	40.00
7	30	1000	40	26.67	40.00
8	25	1000	40	32.00	40.00
9	20	1000	40	40.00	40.00
10	15	1000	40	53.33	40.00

**Table 5:** EDS compositions of the microzones marked in the joint interface shown in Figure 6a.

Element (at%)	Si	O	Al	Possible Phases
Region C	7.35	29.38	57.00	Al_2_O_3_-SiO_2_, Al
Region D	4.70	0.77	91.14	Base Al metal
Region E	21.20	51.77	12.66	Al_2_O_3_-SiO_2_, Al
Region F	7.27	16.33	65.67	Al_2_O_3_-SiO_2_, Al
Region G	5.50	22.42	64.03	Al_2_O_3_-SiO_2_, Al
Region H	0.90	0.67	91.40	Base Al metal

## Data Availability

The raw data supporting the conclusions of this article will be made available by the authors on request.
